# Murine P-glycoprotein on stromal vessels mediates multidrug resistance in intracerebral human glioma xenografts.

**DOI:** 10.1038/bjc.1997.408

**Published:** 1997

**Authors:** Y. Takamiya, Y. Abe, Y. Tanaka, A. Tsugu, M. Kazuno, Y. Oshika, K. Maruo, Y. Ohnishi, O. Sato, H. Yamazaki, H. Kijima, Y. Ueyama, N. Tamaoki, M. Nakamura

**Affiliations:** Department of Neurosurgery, Tokai University School of Medicine, Kanagawa, Japan.

## Abstract

**Images:**


British Journal of Cancer (1997) 76(4),445–450
© 1997 Cancer Research Campaign

### Murine P-glycoprotein on stromal vessels mediates multidrug resistance in intracerebral human glioma xenografts

Y Takamiya[1], Y Abe[2], Y Tanaka[1], A Tsugu[1], M Kazuno[1], Y Oshika[2], K Maruo[3], Y Ohnishi[4], O Sato[1], H Yamazaki[2], H Kijima[2], Y Ueyama[2,4,5], N Tamaoki[2] and M Nakamura[2,5]

Departments of [1]Neurosurgery and [2]Pathology, Tokai University School of Medicine, Bohseidai, Isehara, Kanagawa 259-11; [3]Department of Veterinary Surgery, Faculty of Agriculture, Tokyo University of Agriculture and Technology, 3-5-8 Saiwai, Fuchu, 183, [4]Central Institute for Experimental Animals, Nogawa 1430, Kawasaki-shi, Kanagawa 213; [5]Kanagawa Academy of Science and Technology (KAST), 3-2-1 Sakado, Takatsu, Kawasaki, Kanagawa 213, Japan

**Summary** Human glioma usually shows intrinsic multidrug resistance because of the blood–brain barrier (BBB), in which membrane-associated P-glycoprotein (P-gp), encoded by the human multidrug resistance gene *MDR1*, plays a role. We studied drug sensitivity to vincristine (VCR), doxorubicin (DOX) and nimustine (ACNU) in both intracerebrally and subcutaneously xenotransplanted human glioma. We examined the levels of *MDR1* and murine *mdr3* gene expression in the xenografts by reverse transcriptase polymerase chain reaction and the localization of P-gp by immunohistochemistry. Six of seven subcutaneously transplanted xenografts (scX) were sensitive to the above three drugs. In contrast, all three intracerebrally transplanted human glioma xenografts (icX) were resistant to P-gp-mediated drugs VCR and DOX, but were sensitive to the non-P-gp-mediated drug ACNU. Neither icX nor scX showed any *MDR1* expression. Intracerebrally transplanted human glioma xenografts showed an increased level of murine *mdr3* gene expression, whereas scX showed only faint expression. The localization of P-gp was limited to the stromal vessels in icX by immunohistochemistry, whereas scX expressed no P-gp. Our findings suggest that the P-gp expressed on the stromal vessels in icX is a major contributing factor to multidrug resistance in human glioma in vivo.

**Keywords:** multidrug resistance; P-glycoprotein; human glioma; endothelial cell; blood–brain barrier

Total removal of glioma is clinically difficult as the borders are ill defined. In addition, glioma shows intrinsic multidrug resistance to anti-cancer agents, and chemotherapy for glioma is thus unsuccessful (Edwards et al, 1980; Kornblith et al, 1988). Various mechanisms, including the blood–brain barrier (BBB), have been considered to be involved in the multidrug resistance of glioma.

Certain ultrastructural features, including the absence of fenestration and the presence of tight junctions, are considered to characterize the BBB (Reese et al, 1967; Long, 1970). However, it has been reported (Levin, 1980) that hydrophobic anti-cancer agents such as vinca alkaloids and doxorubicin (DOX) do not easily enter the brain, although other hydrophobic molecules readily passed through the BBB. Thus, it appears that morphological BBB structures cannot completely explain the multidrug resistance in human glioma.

Some studies have indicated that the membrane-associated protein P-glycoprotein (P-gp), encoded by human multidrug resistance gene *MDR1*, is related to the multidrug resistance phenotype in human neoplasms (Chen et al, 1986; 1990; Gros et al, 1986), and that P-gp is a component of the BBB in the normal brain (Tatsuta et al, 1992). We have revealed that, in human glioma, P-gp is expressed not on the tumour cells but on the capillary blood vessels as determined by immunohistochemical and molecular biological methods (Tanaka et al, 1994).

*Received 21 October 1996*
*Revised 14 February 1997*
*Accepted 18 February 1997*

*Correspondence to:* M Nakamura

In our other previous report, almost all the glioma xenografts subcutaneously transplanted in the nude mice were curiously sensitive to P-gp-mediated anti-cancer drugs (vincristine, VCR and DOX) without *MDR1* expression (Abe et al, 1994*a*), whereas these drugs are usually ineffective in clinical cases.

In this study, we performed intracerebral (icX) and subcutaneous (scX) transplantation of human glioma into nude mice to analyse the differences in chemosensitivity to anti-cancer drugs between clinical glioma and scX. We studied the chemosensitivity in vivo and the levels of expression of human *MDR1* and murine *mdr3* (also called *mdr1a*) gene in these glioma xenografts. We also examined the localization of P-gp in these glioma xenografts by immunohistochemistry. We discuss here the mechanisms of intrinsic multidrug resistance of glioma in relation to stromal endothelial P-gp expression in vivo.

### MATERIALS AND METHODS

### Human glioma xenografts and cell lines

Seven human glioma xenografts were established from primary glioma specimens obtained from patients in whom there had been no preceding chemotherapy (Table 1). The xenografts were maintained by serial subcutaneous transplantation in nude mice (BALB/c–nu/nu, Clea Japan, Tokyo). The xenografts were used at 10–20 passages. We obtained xenografts from mice after they had been deeply anaesthetized with chloroform. The drug-sensitive epidermoid carcinoma cell line KB3-1 and its resistant derivative KB8-5 were cultured in Dulbecco's modified Eagle minimal essential medium supplemented with 5% fetal bovine serum at 37°C in a fully humidified 95% air, 5% carbon dioxide atmosphere.

**Table 1** Patient characteristics of primary glioma used for establishment of xenografts

| Xenograft | Age | Sex | Histology | Clinical therapy[a] |
|---|---|---|---|---|
| Epe-1 | 10 | M | EPE | Surg |
| GL-1 | 3 | M | EPE | Surg + Ra |
| GL-2 | 32 | M | GM | Surg + Ra |
| GL-3 | 47 | M | GM | Surg |
| GL-5 | 50 | M | GM | Surg |
| GL-7 | 67 | F | GM | Surg |
| GL-8 | 10 | M | GM | Surg |

EPE, ependymoma; GM, glioblastoma multiforme; Surg, surgical removal; Ra, radiation therapy. [a]Chemotherapy was not performed on these patients.

### In vivo chemosensitivity test

DOX (Kyowa Hakkoh Kogyo, Tokyo), VCR (Shionogi, Osaka) and ACNU (Sankyo, Tokyo) were purchased from the sources shown. All drugs were dissolved in saline and used for in vivo chemosensitivity tests.

We performed in vivo chemosensitivity tests on seven scX (Epe-1, GL-1, GL-2, GL-3, GL-5, GL-7, and GL-8), according to the procedures reported previously (Inaba et al, 1989; Abe et al, 1996*a,b*). The nude mice were used at 6–8 weeks of age in accordance with the animal care guidelines of the Central Institute for Experimental Animals. Six female mice (BALB/c–nu/nu) bearing xenografts (tumour volume: 100–300 $mm^3$) were given the maximum tolerated dose (MTD) of VCR (1.6 mg $kg^{-1}$) or DOX (12 mg $kg^{-1}$). Growth of the tumour xenografts was measured by the relative tumour volume (RV), which was expressed as RV = $V_{14}/V_0$, where $V_{14}$ is the tumour volume at day 14 and $V_0$ is the initial tumour volume when the treatment was started (day 0). The effects of the drugs are represented by RV of the xenografts, and the T/C% values were defined as the ratio of RV of the treated tumour xenografts to controls. Evaluation as 'sensitive' was defined based on statistical significance determined by the Mann–Whitney *U*-test ($P < 0.01$, one-sided).

We also examined in vivo chemosensitivity in three icX (GL-2, GL-5, and GL-8), according to the previously reported method (Ausman et al, 1970; Shapiro et al, 1979; 1981). When the animals showed severe wasting and were apparently moribund owing to intracerebral tumour growth, they were not observed further and the day of sacrifice was recorded to estimate life span according to the UKCCCR guidelines (Workman et al, 1988). Surviving animals were sacrificed at the end of the experiment and sectioned for microscopic examination of tumour foci. The glioma xenograft specimens were removed from nude mice that had been deeply anaesthetized, and were immediately suspended in Ham's F10 culture medium. For intracerebral inoculation, $1 \times 10^6$ cells (in 0.025 ml) were injected percutaneously into the right cerebral hemisphere of nude mice using a 27-gauge needle. Six nude mice bearing the icX were intravenously treated with the MTD of each drug 5 days after inoculation. Drug sensitivity was represented by determining the percentage increase in lifespan (ILS) of the treated nude mice compared with controls. The significance of differences of ILS between treated and control mice was statistically tested by Student's *t*-test.

### Reverse transcriptase polymerase chain reaction (RT-PCR)

The levels of expression of *MDR1* transcripts were determined by the modified reverse transcriptase polymerase chain reaction (RT-PCR) procedure as described previously (Noonan et al, 1990; Abe et al, 1994*b*). Normal human tissues (brain and kidney) were obtained by major surgery after obtaining the patients' informed consent. RT-PCR amplified a 243-bp fragment of *MDR1* cDNA. We estimated *MDR1* expression level in comparison with that of the housekeeping gene $\beta_2$-microglobulin ($\beta_2$m).

We also prepared sets of specific primers for murine *mdr3* (#M; sense, CATGGCTGGATCAGTGTTTCTAGA, residues 3391–3415; antisense, GCAGTGAGTCGATGAACTGGTGGA, residues 3585–3608), and for murine $\beta_2$m (#N; sense, GCAAGGACTGGTCTTTCTATA, residues 3144–3164; antisense, GCATGACAGTATGGCCGAGCC, residues 3223–3243) (Parnes et al, 1982; Gros et al, 1988). RT-PCR with these amprimers revealed a 218-bp segment of murine *mdr3*. The PCR products blotted on a membrane (Zeta Probe, BioRad) were detected by hybridization with synthetic oligonucleotide probes (GCCGTGTCTCATGAGGAGATTGTG, residues 3518–3541, for #M; CTGATACATACGCCTGCAGAG, residues 3217–3240, for #N) labelled with $^{32}$P.

### Immunohistochemistry

P-gp-positive tumour cells were analysed immunohistochemically with the anti-P-gp polyclonal antibody Ab-1 (Oncogene Science) (Toth et al, 1994; Abe et al, 1996*a*). Tumour sections were incubated with Ab-1, peroxidase-conjugated $F(ab')_2$ of donkey anti-rabbit IgG (Amersham), rabbit monoclonal peroxidase–anti-peroxidase complex (Dako) and peroxidase-conjugated $F(ab')_2$ fragments. The products were visualized with 3,3′-diaminobenzidine tetrahydrochloride.

### RESULTS

### In vivo chemosensitivity test

Table 2 summarizes the growth features of the scX, including relative tumour volume, T/C%, and Mann–Whitney *U*-test results ($P < 0.01$, one-sided). All seven scX showed T/C% values significantly less than 35%, and were thus considered to be sensitive to VCR and ACNU. Six of seven scX were also sensitive to DOX (T/C% < 32%). Only one scX (GL-5) showed a relatively high T/C% value to DOX (66%). The glioma scX showed high response rates of 100%, 100% and 86% to VCR, ACNU and DOX respectively. The maximum volume of tumour xenograft did not exceed 10% of the weight of the mouse (data not shown).

The drug sensitivity of icX is shown in Table 3. None of the three icX treated with VCR and DOX showed any significant elongation of the ILS (less than 55%), whereas the icX treated with ACNU showed remarkably high values for the ILS. Significant effects of ACNU on the glioma icX were also confirmed by statistical analysis by Student's *t*-test ($P < 0.01$).

### Expression of *MDR1*

The human *MDR1*-specific amprimers did not amplify murine *mdr* cDNA from normal murine tissues (brain, heart, lung, kidney, liver, spleen), although the sense and antisense primers showed

 © Cancer Research Campaign 1997

**Table 2** In vivo chemotherapy of subcutaneously transplanted glioma xenograft (scX)

| Xenograft drug | Relative tumour volume[a] | | T/C[b] (%) | U-test[c] |
|---|---|---|---|---|
| | Control | Treated | | |
| VCR | | | | |
| Epe-1 | 6.08 ± 2.33 | 0.61 ± 0.23 | 10 | + |
| GL-1 | 3.59 ± 0.57 | 0.37 ± 0.55 | 10 | + |
| GL-2 | 8.48 ± 3.04 | 0.54 ± 0.12 | 6 | + |
| GL-3 | 7.59 ± 3.40 | 0.33 ± 0.14 | 4 | + |
| GL-5 | 4.68 ± 1.19 | 0.43 ± 0.17 | 9 | + |
| GL-7 | 12.29 ± 4.13 | 0.09 ± 0.02 | 1 | + |
| GL-8 | 7.25 ± 1.99 | 0.21 ± 0.11 | 3 | + |
| DOX | | | | |
| Epe-1 | 6.08 ± 2.33 | 1.73 ± 0.34 | 28 | + |
| GL-1 | 3.04 ± 0.92 | 0.97 ± 0.41 | 32 | + |
| GL-2 | 8.48 ± 3.04 | 2.42 ± 0.40 | 29 | + |
| GL-3 | 7.59 ± 3.40 | 1.29 ± 0.18 | 17 | + |
| GL-5 | 4.68 ± 1.19 | 3.10 ± 0.94 | 66 | – |
| GL-7 | 14.31 ± 4.35 | 1.79 ± 1.49 | 13 | + |
| GL-8 | 11.08 ± 3.73 | 2.12 ± 1.08 | 19 | + |
| ACNU | | | | |
| Epe-1 | 6.08 ± 2.33 | 2.11 ± 0.67 | 35 | + |
| GL-1 | 3.96 ± 1.31 | 0.90 ± 0.18 | 23 | + |
| GL-2 | 10.00 ± 3.89 | 1.12 ± 0.12 | 11 | + |
| GL-3 | 10.69 ± 3.54 | 2.85 ± 1.11 | 27 | + |
| GL-5 | 7.12 ± 3.66 | 0.30 ± 0.12 | 4 | + |
| GL-7 | 12.29 ± 4.13 | 0.07 ± 0.03 | 1 | + |
| GL-8 | 7.25 ± 1.99 | 0.46 ± 0.20 | 6 | + |

[a]Relative tumour volume (*RV*), $RV = V_{14}/V_0$, where $V_{14}$ is the tumour volume on day 14 and $V_0$ is the initial tumour volume when the treatment was started (day 0). [b]T/C (%) values defined as the ratio of the *RV* of the treated tumour xenografts to that of controls on day 14 after drug administration. [c]*U*-test, statistical differences were determined by the Mann–Whitney *U*-test ($P < 0.01$, one-sided; +, significant; –, insignificant). VCR, vincristine; DOX, doxorubicin; ACNU, nimustine.

**Figure 1** Human multidrug resistance gene (*MDR1*) expression. *MDR1* (243 bp) and human $\beta_2$-microglobulin (H-$\beta_2$m, 120 bp) were amplified by 26 cycles of PCR with a cDNA reverse transcribed from 500 ng of the total cellular RNA. (**A**) *MDR1* expression in the human and murine normal tissues. Lane 1, human kidney; lane 2, murine kidney; lane 3, human brain; lane 4, murine brain. (**B**) *MDR1* expression in the human glioma transplanted subcutaneously (scX) and intracerebrally (icX). Lane 1, KB8-5; lane 2, KB 3-1; lane 3, GL-2 (scX); lane 4, GL-3 (scX); lane 5, GL-5 (scX); lane 6, GL-8 (scX); lane 7, GL-2 (icX); lane 8, GL-5 (icX); lane 9, GL-8 (icX)

**Table 3** In vivo chemotherapy of intracerebrally transplanted glioma xenograft (icX)

| Xenograft drug | Survival days | | ILS (%) |
|---|---|---|---|
| | Control | Treated | |
| VCR | | | |
| GL-2 | 32.2 ± 6.1 | 41.5 ± 2.8 | 29 |
| GL-5 | 34.2 ± 4.2 | 45.2 ± 3.1 | 32 |
| GL-8 | 28.0 ± 3.6 | 32.3 ± 2.1 | 15 |
| DOX | | | |
| GL-2 | 28.5 ± 4.9 | 44.3 ± 9.2 | 55 |
| GL-5 | 37.7 ± 4.4 | 39.5 ± 6.1 | 5 |
| GL-8 | 28.0 ± 3.6 | 36.7 ± 6.9 | 31 |
| ACNU | | | |
| GL-2 | 28.5 ± 4.9 | 97.2 ± 5.9 | 241[a] |
| GL-5 | 41.7 ± 5.6 | 99.0 ± 4.4 | 137[a] |
| GL-8 | 28.0 ± 3.6 | 80.5 ± 12.8 | 188[a] |

[a]Significant by Student's *t*-test ($P < 0.01$). ILS, increase in lifespan; VCR, vincristine; DOX, doxorubicin; ACNU, nimustine.

**Table 4** *MDR1*, *mdr3* gene and P-gp expression in icX and scX human glioma

| Xenograft | *MDR1*[a] | *mdr3*[b] | P-gp[c] |
|---|---|---|---|
| icX | 0/3 | 3/3 | 3/3 |
| scX | 0/7 | 3/7[d] | 0/7 |

[a,b]Human multidrug resistance gene (*MDR1*) and murine mdr3 gene expression were detected by reverse transcriptase polymerase chain reaction assay. [c]The expression of murine *P*-glycoprotein (P-gp) was detected only on endothelial cells by immunohistochemical analysis. [d]The levels of *mdr3* gene expression in three scX were very faint (see Figure 2C). scX, xenografts transplanted intracerebrally; icX, xenografts transplanted subcutaneously.

96% and 75% homology, respectively, with the murine *mdr3* gene (Figure 1A). We thus avoided amplification of murine *mdr* gene transcripts contaminating the tumour xenografts.

Neither human glioma scX nor icX expressed *MDR1* (Table 4, Figure 1B). Colon and pancreatic cancer xenografts uniformly retained levels of *MDR1* gene expression similar to those in the original carcinomas (data not shown).

### Expression of murine mdr3 gene

Murine *mdr3*-specific amprimers did not amplify the human *MDR1* cDNA from normal human tissues (brain and kidney, Figure 2A), although both sense and antisense primers revealed 67% homology with the human *MDR1* gene. We thus specifically determined murine *mdr3* gene transcripts with these amprimers. The amount of the PCR product of murine *mdr3* was proportional to the initial template cDNA reverse transcribed from mRNA under our experimental conditions.

We quantitatively analysed the RT-PCR of *mdr3* mRNA in murine brain, according to the modified procedures reported previously (Abe et al, 1993; 1994*b*). The specific *mdr3* products were also amplified exponentially by 22–32 PCR cycles (data not shown). The results suggested that *mdr3* gene expression can be semiquantified by 28 cycles of RT-PCR with 500 ng of RNA. The icX showed significantly stronger *mdr3* expression than normal murine brain (Figure 2B), whereas three of seven scX showed only faint levels of *mdr3* expression (Figure 2C).

© Cancer Research Campaign 1997

**Figure 2** Murine multidrug-resistance gene (mdr3) expression. Specific PCR products for mdr3 (218 bp) and for murine β2-microgloburine (M-$\beta_2$m, 100 bp) were amplified by 26 cycles of PCR with a cDNA reverse transcribed from 500 ng. (**A**) *mdr3* gene expression in the normal human (H) and murine (M) tissues. Lane 1, M-kidney; lane 2, H-kidney; lane 3, M-brain; lane 4, H-brain; lane 5, M-liver; lane 6, M-spleen; lane 7, M-heart; lane 8, M-skin. (**B**) The mdr3 gene expression in the human glioma transplanted intracerebrally (icX). Lane 1, GL-2 (icX); lane 2, M-brain; lane 3, GL-5 (icX); lane 4, GL-8 (icX). (**C**) *mdr3* gene expression in the human glioma transplanted subcutaneously (scX). Lane 1, M-brain; lane 2, GL-2 (scX); lane 3, GL-5 (scX); lane 4, GL-8 (scX)

**Figure 3** Localization of P-gp: immunohistochemical analysis was performed with anti-P-gp polyclonal antibody Ab-1. Positive staining was observed at the vessels in the intracerebrally (icX) transplanted glioma xenografts (GL-8, right upper side) and the extraneoplastic brain tissue (left lower side) (x 100)

### Localization of P-gp

We examined the localization of P-gp in human glioma of icX and scX. The localization of P-gp was limited to the stromal vessels in icX by immunohistochemistry (Figure 3), while the scX expressed no P-gp (Table 4). P-gp was produced specifically on the capillary blood vessels, whereas neither tumour nor normal glial cells showed P-gp expression.

### DISCUSSION

Human glioma usually shows intrinsic multidrug resistance to various anti-cancer agents. The multidrug-resistance phenomenon of glioma has been explained by the presence of the BBB on the stromal vessels. Chemotherapy with nitrosoureas, such as BCNU and ACNU, has been shown to be effective in glioma, whereas such anti-cancer drugs as VCR and DOX were not successful (Edwards et al, 1980; Kornblith et al, 1988). Previous studies have indicated that glioma xenografts, transplanted subcutaneously into host animals, were uniformly sensitive to such anti-cancer drugs, as VCR, DOX and ACNU in vivo (Maruo et al, 1990; Abe et al, 1994*a*).

In this study, we examined the drug sensitivity of human glioma scX and icX in vivo to examine the above discrepancy between the clinical drug sensitivity of glioma and the experimental drug sensitivity of scX. The icX was relatively resistant to VCR and DOX (ILS% ≤ 55%), and was sensitive only to ACNU (ILS% ≥ 137%). The icX reflected the clinical drug sensitivity of glioma. However, scX were sensitive to all three anti-cancer drugs (T/C% ≤ 66%).

Several studies have indicated that P-gp constitutes part of the BBB (Thiebaut et al, 1987), whereas some studies have shown that the expression of P-gp is limited to stromal capillary endothelial cells (Cordon-Cardo et al, 1989; Sugawara et al, 1990; Hegmann et al, 1992, Tanaka et al, 1994). However, others have suggested that P-gp overexpression in glioma cells contributes to MDR (Matsumoto et al, 1991; Nabors et al, 1991; Becker et al, 1991). Although these studies employed immunohistochemical methods using anti-P-gp monoclonal antibodies, the localization and apparent function of P-gp in the normal brain and glioma were in conflict. P-gp or *MDR1* gene overexpression in solid tumour cells is generally thought to be an essential mechanism of multidrug resistance to hydrophobic anti-cancer agents (Gottesman et al, 1991). In the present study, neither the scX nor icX of human glioma revealed *MDR1* expression by the RT-PCR assay. Murine *mdr3* expression was enhanced in the icX compared with the normal murine brain, whereas the scX did not show significant enhancement of *mdr3* expression. By immunohistochemical analysis, we confirmed limited P-gp expression not on tumour cells, but on capillary blood vessel walls.

Replacement of the human cerebral stroma, including blood vessels, by the murine subcutaneous stroma resulted in the loss of *MDR1* expression in the human glioma xenografts (Tanaka et al, 1994). This molecular biological finding in glioma xenografts is consistent with the limited localization on the endothelial cells described above.

Murine *mdr3* expression was enhanced in the icX compared with the normal murine brain, whereas the scX did not show significant enhancement of *mdr3* expression. Schinkel et al (1994) reported that the expression of the murine *mdr3* gene was preferentially related to multidrug resistance in murine brain. We confirmed that the level of *mdr3* gene expression was higher than

© Cancer Research Campaign 1997

that of murine *mdrl* (also called *mdrlb*) in normal murine brain and in human glioma xenografts (data not shown). Our results support the idea that murine P-gp, encoded by *mdr3*, contributed to the multidrug resistance of the human glioma icX in vivo.

The reversal of P-gp function produced by anti-P-gp monoclonal antibody, or by various compounds such as calcium channel antagonists, calmodulin inhibitors and cyclosporins, has overcome multidrug resistance in many tumours in vitro and in vivo (Boesch et al, 1991; Kadam et al, 1992; Mickisch et al, 1992; Miyamoto et al, 1993). The results presented here suggest that glioma cells are themselves sensitive to VCR, DOX and ACNU, and that circumvention of the BBB-like function of P-gp could allow successful chemotherapy with these conventional anticancer agents. The scX and icX systems of human glioma appear to be useful models for study of the intrinsic multidrug resistance of glioma in vivo, and for studies of ways in which this multidrug resistance can be overcome.

We did not examine the glioma or glioma xenografts after chemotherapy, and it is unclear whether the overexpression of P-gp is inducible in glioma tumour cells. Further analysis of P-gp expression in xenografts after experimental treatment will provide more information on the acquired multidrug resistance of glioma in vivo.

### ACKNOWLEDGEMENTS

This work was supported in part by Grants-in-Aid for Cancer and Scientific Research from the Ministry of Education, Science and Culture of Japan (MN, 06670206, YU 05670210 and 07680921, NT 07457588), by Tokai University School of Medicine Research Aid (MN, YU, HY) and by a Grant-in Aid to the DNA Diagnosis Project from Tokai University School of Medicine (MN). We also thank Mr Y Tada Mr J Itoh, Ms M Yoshimura, Ms K Murata and Ms R Saegusa, for their excellent assistance.

### REFERENCES

Abe Y, Nakamura M, Saegusa R, Ueyama Y, Ogata T and Tamaoki N (1993) In vivo acquired drug resistance and multidrug resistance gene (MDR1) expression in the KB carcinoma cell line xenotransplanted in nude mice. *Tokai J Exp Clin Med* **18**: 99–106

Abe Y, Nakamura M, Ohnishi Y, Inaba M, Ueyama Y and Tamaoki N (1994*a*) Multidrug resistance gene (MDR1) expression in human tumor xenografts. *Int J Oncol* **5**: 1285–1292

Abe Y, Nakamura M, Ota E, Ozeki U, Tamai S, Inoue H, Ueyama Y, Ogata T and Tamaoki N (1994*b*) Expression of the multidrug resistance gene (MDR1) in non-small cell lung cancer. *Jpn J Cancer Res* **85**: 536–541

Abe Y, Yamazaki H, Oshika Y, Suto R, Tsugu A, Ota E, Satoh H, Ohnishi Y, Yanagawa T, Ueyama Y, Tamaoki N and Nakamura M (1996*a*) Advantage of in vivo chemosensitivity assay to detect vincristine-resistance in a human epidermoid carcinoma xenograft. *Anticancer Res* **16**: 729–734

Abe Y, Ohnishi Y, Yoshimura M, Ota E, Ozeki Y, Oshika Y, Tokunaga T, Yamazaki H, Ueyama Y, Ogata T, Tamaoki N and Nakamura M (1996*b*) P-glycoprotein-mediated acquired multidrug resistance of human lung cancer cells in vivo. *Br J Cancer* **74**: 1929–1934

Ausman JI, Shapiro WR and Rall DP (1970) Studies on the chemotherapy of experimental brain tumours: Development of an experimental model. *Cancer Res* **30**: 2394–2400

Becker I, Becker KF, Meyerman R and Hollt V (1991) The multidrug-resistance gene MDR1 is expressed in human glial tumours. *Acta Neuropathol* **82**: 516–519

Boesch D, Gaverriaux C, Jachez B, Pourtier-Manzanedo A, Bollinger P and Loor F (1991) In vivo circumvention of P-glycoprotein-mediated multidrug resistance of tumour cells with SDZ PSC 833. *Cancer Res* **51**: 4226–4233

Chen C, Chin JE, Ueda K, Clark DP, Pastan I, Gottesman MN and Roninson IB (1986) Internal duplication and homology with bacterial transport proteins in the MDR1 (P-glycoprotein) gene from multidrug-resistant human cells. *Cells* **47**: 381–389

Chen C, Clark D, Ueda K, Pastan I, Gottesman MM and Roninson IB (1990) Genomic organization of the human multidrug resistance (*MDR1*) gene and origin of P-glycoproteins. *J Biol Chem* **265**: 506–514

Cordon-Cardo C, O'Brien JP, Casals D, Rittman-Grauer L, Biedler JL, Melamed MR and Bertino JR (1989) Multidrug-resistance gene (P-glycoprotein) is expressed by endothelial cells at blood-brain barrier sites. *Proc Natl Acad Sci USA* **86**: 695–698

Edward MS, Levin VA and Wilson CB (1980) Brain tumour chemotherapy: an evaluation of agents in current use for phase II and III. *Cancer Treatment Reports* **64**: 1179–1205

Gottesman MM, Goldstein L, Fojo A, Galski H and Pastan I (1991) Expression of the multidrug resistant gene in human cancer. In *Molecular and Cellular Biology of Multidrug Resistance in Tumor Cells*. Robinson IB (ed.),pp. 291–291–300. Plenum: New York

Gros P, Neriah YB, Croop JM and Houseman DE (1986) Isolation and expression of a complementary DNA that confers multidrug resistance. *Nature* **323**: 728–731

Gros P, Raymond M, Bell J and Housman D (1988) Cloning and characterization of a second member of the mouse mdr gene family. *Mol Cell Biol* **8**: 2770–2778

Hegman EJ, Bauer HC and Kerbel RS (1992) Expression and functional activity of p-glycoprotein in cultured cerebral capillary endothelial cells. *Cancer Res* **52**: 6969–6975

Inaba M, Kobayashi T, Tashiro T, Sakurai Y, Maruo K, Ohnishi Y, Ueyama Y and Nomura T (1989) Evaluation of antitumour activity in a human breast tumour/nude mouse model with a special emphasis on treatment dose. *Cancer* **64**: 1577–1582

Kadam S, Maus M, Podding J, Schmidt S, Rasmussen R, Novosad E, Plattner J and McAlpine J (1992) Reversal of multidrug resistance by two novel indole derivatives. *Cancer Res* **52**: 4735–4740

Kornblith PL and Walker M (1988) Chemotherapy for malignant gliomas. *J Neurosurg* **68**: 1–17

Levin VA (1980) Relationship of octanol/water partition coefficient and molecular weight to rat brain capillary permeability. *J Med Chem* **23**: 682–684

Long DM (1970) Capillary ultrastructure and the blood-brain barrier in human malignant brain tumours. *J Neurosurg* **32**: 127–144

Maruo K, Ueyama Y, Inaba M, Emura R, Ohnishi Y, Nakamura O, Sato O and Nomura T (1990) Responsiveness of subcutaneous human glioma xenografts to various anticancer agents. *Anticancer Res* **10**: 209–212

Matsumoto T, Tani E, Kaba K, Kochi N, Shindo H, Yamamoto Y, Sakamoto H and Furuyama J (1990) Amplification and expression of multidrug resistance gene in human glioma cell lines. *J Neurosurg* **72**: 96–101

Mickisch GM, Pai LH, Gottesman MM and Pastan I (1992) Monoclonal antibody MRK16 reverses the multidrug resistance of multidrug-resistant transgenic mice. *Cancer Res* **52**: 4427–4432

Miyamoto K, Inoko K, Wakusawa S, Kajita S, Hasegawa K, Takagi K and Koyama M (1993) Inhibition of multidrug resistance by a new starosporine derivative, NA-382, in vitro and in vivo. *Cancer Res* **53**: 1555–1559

Nabors MW, Griffin CA, Zehnbauer BA, Hruban RH, Phillips PC, Grossoman SA, Bren H and Colbin OM (1991) Multidrug resistance (MDR1) gene expression in human brain tumours. *J Neurosurg* **75**: 941–946

Noonan KE, Beck C, Holzmayer TA, Chin JE, Wunder JS, Andrulis IL, Gazdar AF, Willman CL, Griffith B, Von Hoff DD and Roninson IB (1991) Quantitative analysis of MDR1 (multidrug resistance) gene expression in human tumours by polymerase chain reaction. *Proc Natl Acad Sci USA* **87**: 7160–7164

Parnes JR and Seidman JG (1982) Structures of wild-type and mutant mouse β2-microglobulin genes. *Cell* **29**: 661–669

Reese TS and Karnovsky MJ (1967) The structural localization of a blood-brain barrier to exogenous peroxidase. *J Cell Biol* **34**: 207–217

Schinkel AH, Smit JJM, Tellingen O, Beijnen JH, Wagenaar E, Deemter L, Mol CAAM, Valk MA, Robanus-Maandag EC, Riele HPJ, Berns AJM and Borst P (1994) Disruption of the mouse mdr1a P-glycoprotein gene leads to a deficiency in the blood-brain barrier and to increased sensitivity to drugs. *Cell* **77**: 491–502

Shapiro WR, Basler GA, Chernik NL and Posner JB (1979) Human brain tumour transplantation into nude mice. *J Natl Cancer Inst* **62**: 447–453

Shapiro WR, Yung WA, Basler GA and Shapiro JR (1981) Heterogeneous response to chemotherapy of human gliomas grown nude mice and as clones in vitro. *Cancer Treat Rep* **65** (suppl. 2): 55–59

© Cancer Research Campaign 1997 

Sugawara I, Hamada H, Tsuruo T and Mori S (1990) Specialized localization of P-glycoprotein recognized by MRK 16 monoclonal antibody in endothelial cells of the brain and spinal cord. *Jpn J Cancer Res* **81**: 727–730

Tanaka Y, Abe Y, Tsugu A, Takamiya Y, Akatsuka A, Tsuruo T, Yamazaki H, Ueyama Y, Sato O, Tamaoki N and Nakamura M (1994) Ultrastructural localization of P-glycoprotein on capillary endothelial cells in human gliomas. *Virchows Arch* **425**: 133–138

Tatsuta T, Naito M, Oh-Hara T, Sugawara I and Tsuruo T (1992) Functional involvement of P-glycoprotein in blood-brain barrier. *J Biolog Chem* **267**: 20383–20391

Thiebaut F, Tsuruo T, Hamada H, Gottesman MM, Pastan I and Willingham MC (1987) Cellular localization of the multidrug-resistance gene product P-glycoprotein in normal human tissues. *Proc Natl Acad Sci USA* **84**: 7735–7738

Toth K, Vaughan MN, Slocum HK, Arredondo MA, Takata H, Baker RM and Rustum YM (1994) New immunohistochemical 'Sandwich' staining method for mdr1 P-glycoprotein detection with JSB-1 monoclonal antibody in formalin-fixed, paraffin-embedded human tissues. *Am J Pathol* **144**: 227–236

Workman P, Balmain A, Hickman JA, McNally NJ, Mitchison NA, Pierepoint CG, Raymond R, Rowlatt C, Stephens TC and Wallace J (1988) UKCCCR guidelines for the welfare of animals in experimental neoplasia. *Br J Cancer* **58**: 109–113

© Cancer Research Campaign 1997


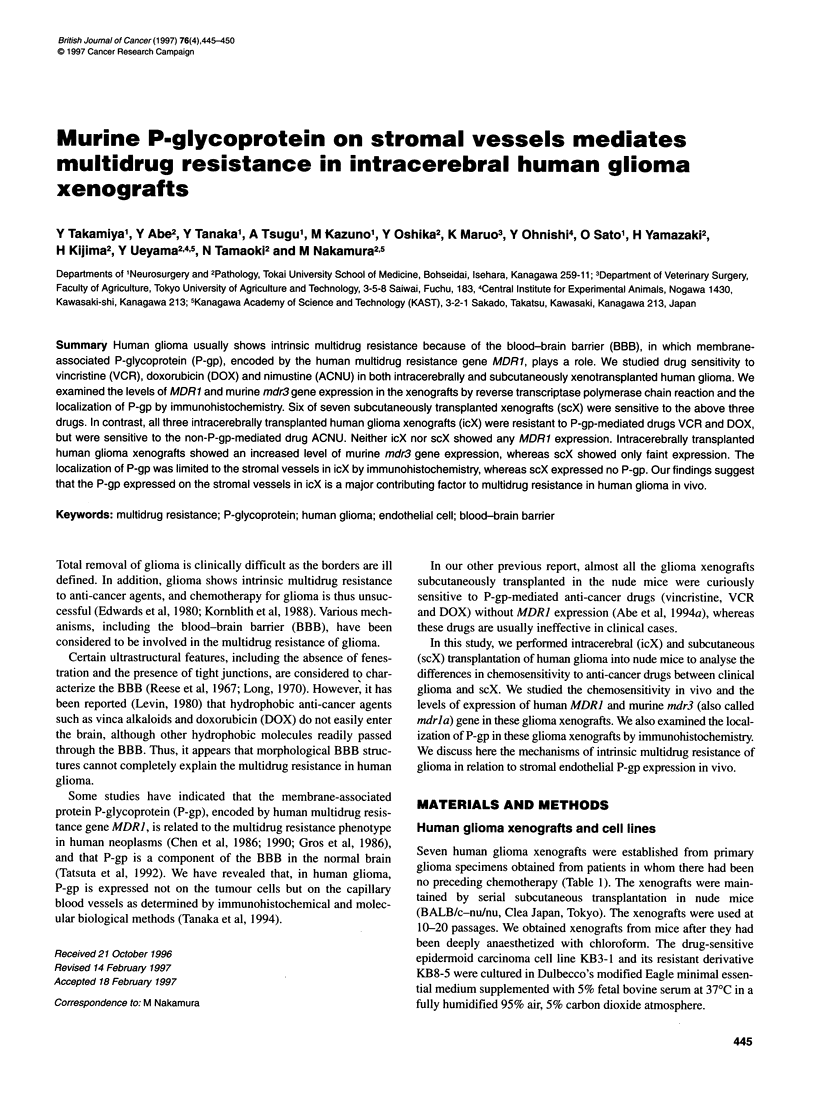

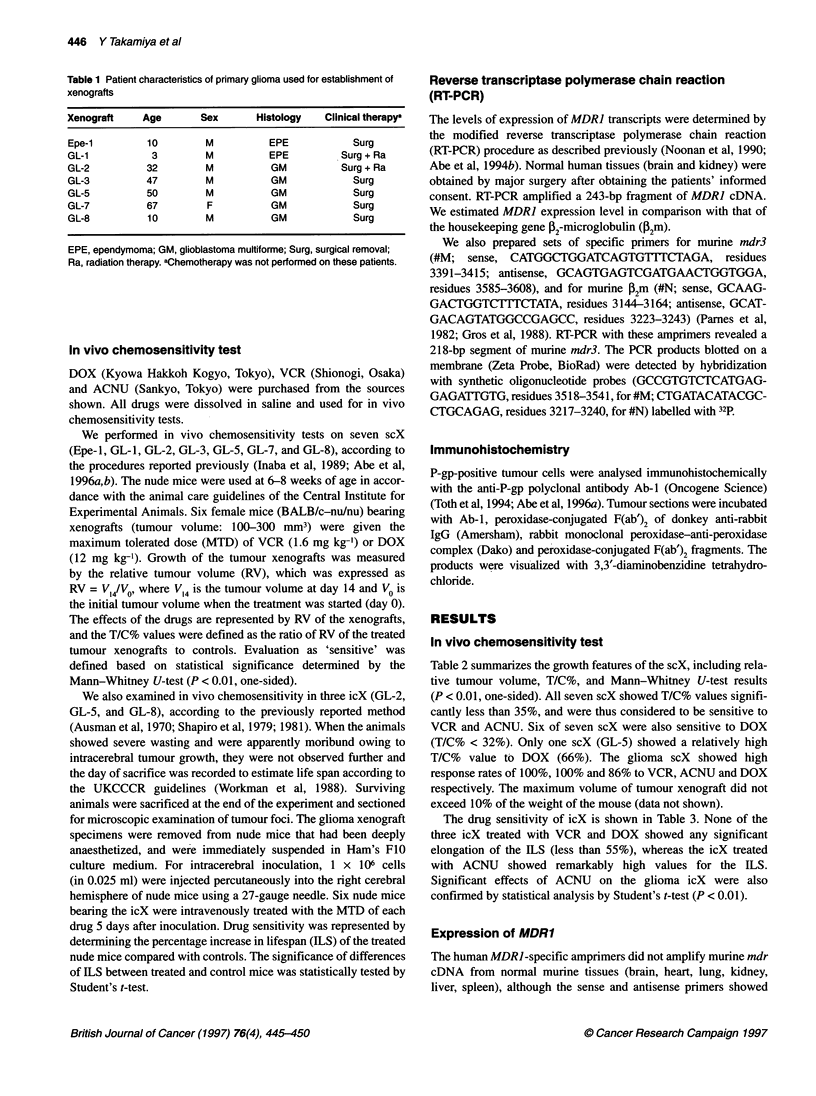

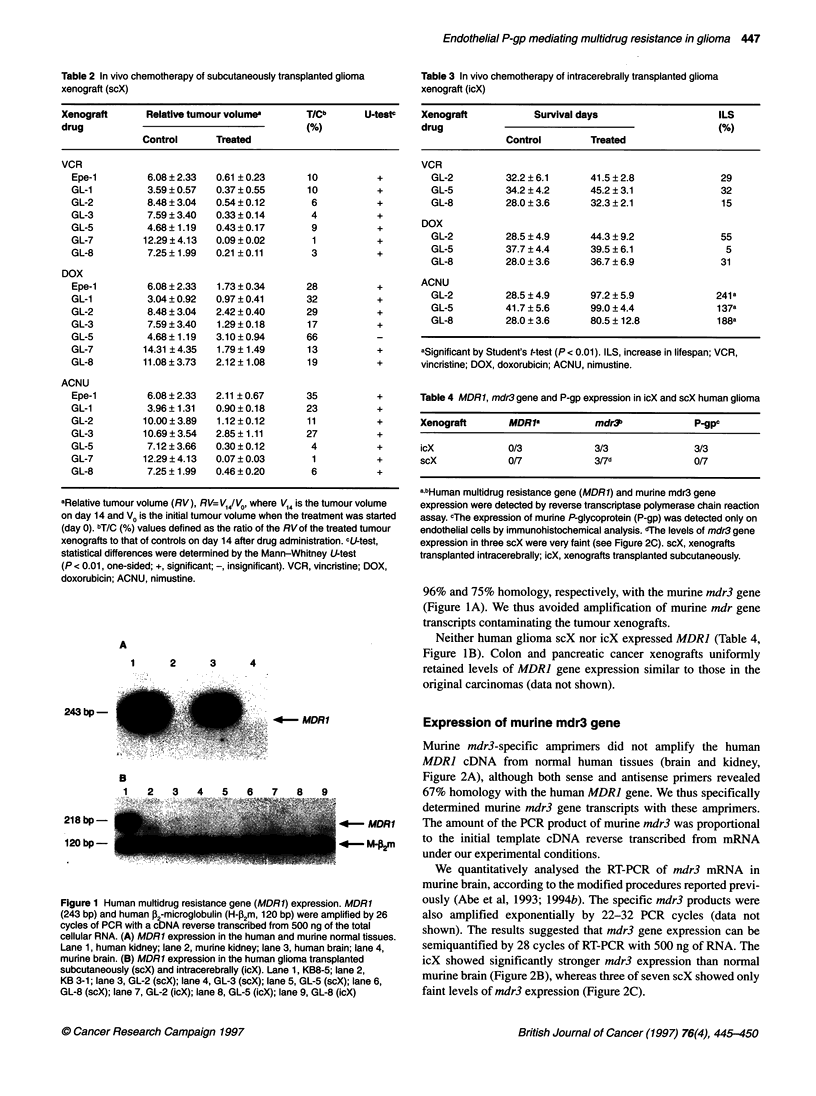

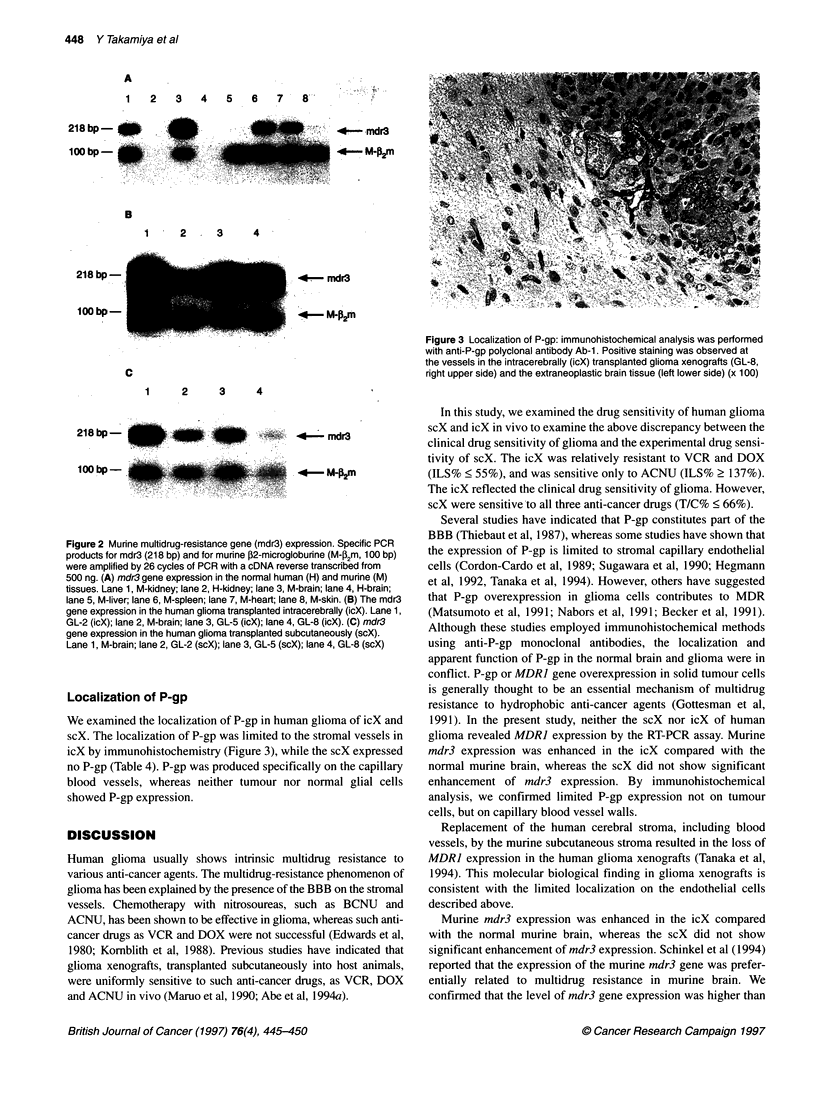

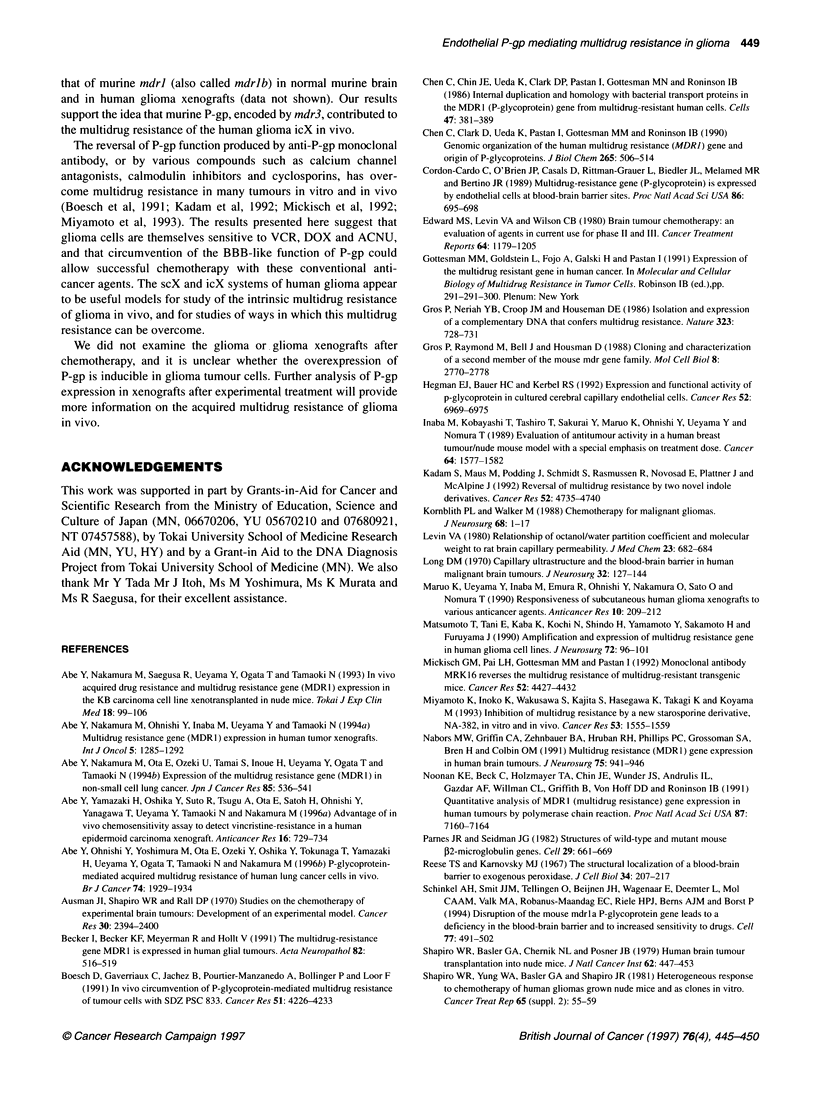

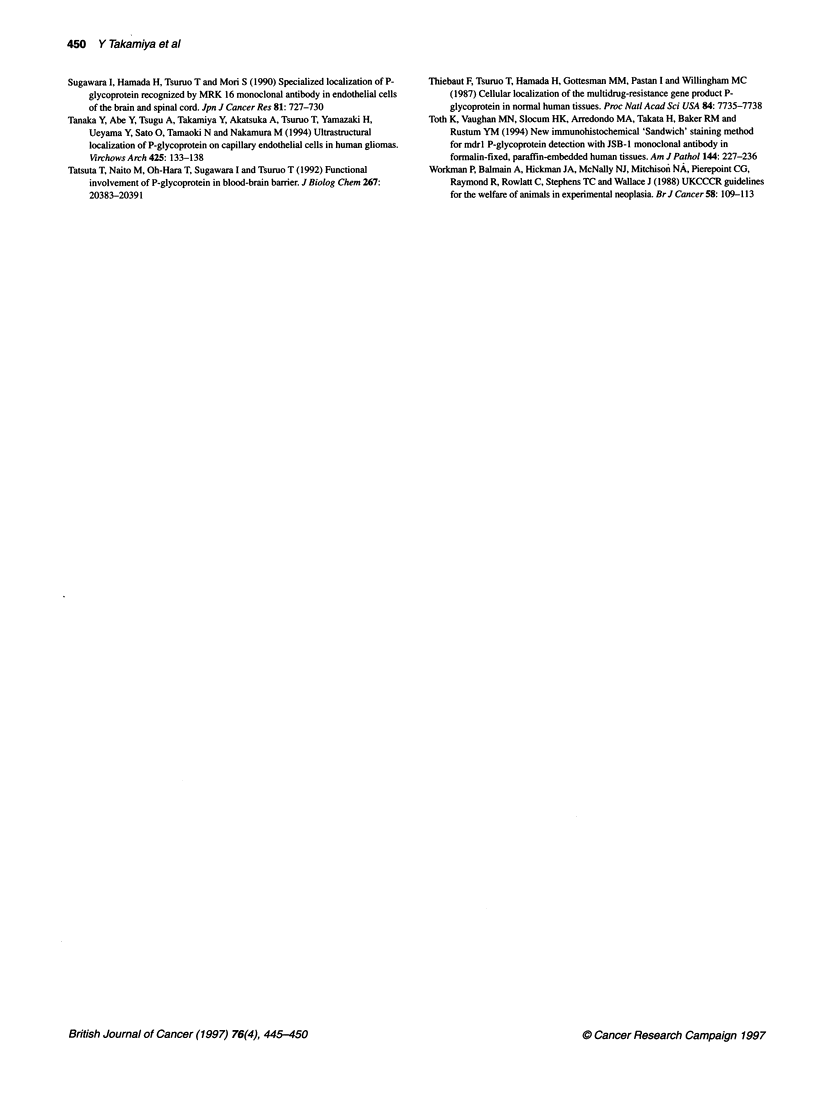


## References

[OCR_00544] Abe Y., Nakamura M., Saegusa R., Ueyama Y., Ogata T., Tamaoki N. (1993). In vivo acquired drug resistance and multidrug resistance gene (MDR1) expression in the KB carcinoma cell line xenotransplanted in nude mice.. Tokai J Exp Clin Med.

[OCR_00566] Abe Y., Ohnishi Y., Yoshimura M., Ota E., Ozeki Y., Oshika Y., Tokunaga T., Yamazaki H., Ueyema Y., Ogata T. (1996). P-glycoprotein-mediated acquired multidrug resistance of human lung cancer cells in vivo.. Br J Cancer.

[OCR_00560] Abe Y., Yamazaki H., Oshika Y., Suto R., Tsugu A., Ota E., Satoh H., Ohnishi Y., Yanagawa T., Ueyama Y. (1996). Advantage of in vivo chemosensitivity assay to detect vincristine-resistance in a human epidermoid carcinoma xenograft.. Anticancer Res.

[OCR_00572] Ausman J. I., Shapiro W. R., Rall D. P. (1970). Studies on the chemotherapy of experimental brain tumors: development of an experimental model.. Cancer Res.

[OCR_00577] Becker I., Becker K. F., Meyermann R., Höllt V. (1991). The multidrug-resistance gene MDR1 is expressed in human glial tumors.. Acta Neuropathol.

[OCR_00582] Boesch D., Gavériaux C., Jachez B., Pourtier-Manzanedo A., Bollinger P., Loor F. (1991). In vivo circumvention of P-glycoprotein-mediated multidrug resistance of tumor cells with SDZ PSC 833.. Cancer Res.

[OCR_00587] Chen C. J., Chin J. E., Ueda K., Clark D. P., Pastan I., Gottesman M. M., Roninson I. B. (1986). Internal duplication and homology with bacterial transport proteins in the mdr1 (P-glycoprotein) gene from multidrug-resistant human cells.. Cell.

[OCR_00593] Chen C. J., Clark D., Ueda K., Pastan I., Gottesman M. M., Roninson I. B. (1990). Genomic organization of the human multidrug resistance (MDR1) gene and origin of P-glycoproteins.. J Biol Chem.

[OCR_00598] Cordon-Cardo C., O'Brien J. P., Casals D., Rittman-Grauer L., Biedler J. L., Melamed M. R., Bertino J. R. (1989). Multidrug-resistance gene (P-glycoprotein) is expressed by endothelial cells at blood-brain barrier sites.. Proc Natl Acad Sci U S A.

[OCR_00604] Edwards M. S., Levin V. A., Wilson C. B. (1980). Brain tumor chemotherapy: an evaluation of agents in current use for phase II and III trials.. Cancer Treat Rep.

[OCR_00615] Gros P., Ben Neriah Y. B., Croop J. M., Housman D. E. (1986). Isolation and expression of a complementary DNA that confers multidrug resistance.. Nature.

[OCR_00620] Gros P., Raymond M., Bell J., Housman D. (1988). Cloning and characterization of a second member of the mouse mdr gene family.. Mol Cell Biol.

[OCR_00625] Hegmann E. J., Bauer H. C., Kerbel R. S. (1992). Expression and functional activity of P-glycoprotein in cultured cerebral capillary endothelial cells.. Cancer Res.

[OCR_00630] Inaba M., Kobayashi T., Tashiro T., Sakurai Y., Maruo K., Ohnishi Y., Ueyama Y., Nomura T. (1989). Evaluation of antitumor activity in a human breast tumor/nude mouse model with a special emphasis on treatment dose.. Cancer.

[OCR_00637] Kadam S., Maus M., Poddig J., Schmidt S., Rasmussen R., Novosad E., Plattner J., McAlpine J. (1992). Reversal of multidrug resistance by two novel indole derivatives.. Cancer Res.

[OCR_00642] Kornblith P. L., Walker M. (1988). Chemotherapy for malignant gliomas.. J Neurosurg.

[OCR_00646] Levin V. A. (1980). Relationship of octanol/water partition coefficient and molecular weight to rat brain capillary permeability.. J Med Chem.

[OCR_00650] Long D. M. (1970). Capillary ultrastructure and the blood-brain barrier in human malignant brain tumors.. J Neurosurg.

[OCR_00654] Maruo K., Ueyama Y., Inaba M., Emura R., Ohnishi Y., Nakamura O., Sato O., Nomura T. (1990). Responsiveness of subcutaneous human glioma xenografts to various antitumor agents.. Anticancer Res.

[OCR_00659] Matsumoto T., Tani E., Kaba K., Kochi N., Shindo H., Yamamoto Y., Sakamoto H., Furuyama J. (1990). Amplification and expression of a multidrug resistance gene in human glioma cell lines.. J Neurosurg.

[OCR_00664] Mickisch G. H., Pai L. H., Gottesman M. M., Pastan I. (1992). Monoclonal antibody MRK16 reverses the multidrug resistance of multidrug-resistant transgenic mice.. Cancer Res.

[OCR_00669] Miyamoto K., Inoko K., Wakusawa S., Kajita S., Hasegawa T., Takagi K., Koyama M. (1993). Inhibition of multidrug resistance by a new staurosporine derivative, NA-382, in vitro and in vivo.. Cancer Res.

[OCR_00674] Nabors M. W., Griffin C. A., Zehnbauer B. A., Hruban R. H., Phillips P. C., Grossman S. A., Brem H., Colvin O. M. (1991). Multidrug resistance gene (MDR1) expression in human brain tumors.. J Neurosurg.

[OCR_00679] Noonan K. E., Beck C., Holzmayer T. A., Chin J. E., Wunder J. S., Andrulis I. L., Gazdar A. F., Willman C. L., Griffith B., Von Hoff D. D. (1990). Quantitative analysis of MDR1 (multidrug resistance) gene expression in human tumors by polymerase chain reaction.. Proc Natl Acad Sci U S A.

[OCR_00687] Parnes J. R., Seidman J. G. (1982). Structure of wild-type and mutant mouse beta 2-microglobulin genes.. Cell.

[OCR_00691] Reese T. S., Karnovsky M. J. (1967). Fine structural localization of a blood-brain barrier to exogenous peroxidase.. J Cell Biol.

[OCR_00695] Schinkel A. H., Smit J. J., van Tellingen O., Beijnen J. H., Wagenaar E., van Deemter L., Mol C. A., van der Valk M. A., Robanus-Maandag E. C., te Riele H. P. (1994). Disruption of the mouse mdr1a P-glycoprotein gene leads to a deficiency in the blood-brain barrier and to increased sensitivity to drugs.. Cell.

[OCR_00703] Shapiro W. R., Basler G. A., Chernik N. L., Posner J. B. (1979). Human brain tumor transplantation into nude mice.. J Natl Cancer Inst.

[OCR_00707] Shapiro W. R., Yung W. A., Basler G. A., Shapiro J. R. (1981). Heterogeneous response to chemotherapy of human gliomas grown in nude mice and as clones in vitro.. Cancer Treat Rep.

[OCR_00716] Sugawara I., Hamada H., Tsuruo T., Mori S. (1990). Specialized localization of P-glycoprotein recognized by MRK 16 monoclonal antibody in endothelial cells of the brain and the spinal cord.. Jpn J Cancer Res.

[OCR_00721] Tanaka Y., Abe Y., Tsugu A., Takamiya Y., Akatsuka A., Tsuruo T., Yamazaki H., Ueyama Y., Sato O., Tamaoki N. (1994). Ultrastructural localization of P-glycoprotein on capillary endothelial cells in human gliomas.. Virchows Arch.

[OCR_00728] Tatsuta T., Naito M., Oh-hara T., Sugawara I., Tsuruo T. (1992). Functional involvement of P-glycoprotein in blood-brain barrier.. J Biol Chem.

[OCR_00733] Thiebaut F., Tsuruo T., Hamada H., Gottesman M. M., Pastan I., Willingham M. C. (1987). Cellular localization of the multidrug-resistance gene product P-glycoprotein in normal human tissues.. Proc Natl Acad Sci U S A.

[OCR_00738] Tóth K., Vaughan M. M., Slocum H. K., Arredondo M. A., Takita H., Baker R. M., Rustum Y. M. (1994). New immunohistochemical "sandwich" staining method for mdr1 P-glycoprotein detection with JSB-1 monoclonal antibody in formalin-fixed, paraffin-embedded human tissues.. Am J Pathol.

[OCR_00744] (1988). UKCCCR guidelines for the welfare of animals in experimental neoplasia.. Br J Cancer.

